# Systematic Evaluation of Strain Rate and Environmental Conditions Effects on Stress Corrosion Cracking of an Al-Cu Alloy

**DOI:** 10.3390/ma19112414

**Published:** 2026-06-05

**Authors:** Sergio Lorenzi, Lorenzo Nani, Samuel Ferrari, Mattia Locatelli, Luca Gritti, Sara Bocchi, Marina Cabrini

**Affiliations:** 1 Department of Engineering and Applied Sciences, University of Bergamo, 24044 Dalmine, Italy; sergio.lorenzi@unibg.it (S.L.); samuel.ferrari@unibg.it (S.F.); mattia.locatelli@unibg.it (M.L.); luca.gritti1@unibg.it (L.G.); marina.cabrini@unibg.it (M.C.); 2 National Interuniversity Consortium of Materials Science and Technology (INSTM), 50121 Firenze, Italy; 3 Department of Management Information and Production Engineering, University of Bergamo, 24044 Dalmine, Italy; sara.bocchi@unibg.it

**Keywords:** aluminum alloys, stress corrosion cracking, environmentally assisted cracking, intergranular corrosion, corrosion, slow strain rate

## Abstract

**Highlights:**

- Strain rate and relative humidity strongly influence SCC susceptibility.
- Pre-soaking localized attacks are necessary for SCC initiation.
- Strain rates below 10^−5^ s^−1^ markedly increase SCC damage.
- IGC susceptibility drives predominant intergranular fracture.
- Critical environmental–mechanical window for SCC testing was identified.

**Abstract:**

The aim of this study is to comprehensively investigate and quantify the effect of strain rate (SR) and environmental parameters on the stress corrosion cracking (SCC) behavior of a high-strength, aluminum–copper alloy. Slow strain rate (SSR) tests were carried out in air at 25 °C, over a SR range from 10^−4^ to 10^−7^ s^−1^ and controlled relative humidity (RH) between 40% and 80%. The influence of the pre-soaking period in 3.5 wt.% NaCl solution was also assessed. A major effect of pre-soaking was identified, as it was necessary for the onset of SCC. Increasing RH over 40% and decreasing SR below 10^−5^ s^−1^ significantly intensified SCC susceptibility, leading to ductility loss up to 84%. SSR test results were supported by microstructural investigations, with particular emphasis on the role of second phases. Their electrochemical activity was examined by scanning Kelvin probe force microscopy (SKPFM), while intergranular corrosion (IGC) susceptibility was evaluated according to the ISO 11846 standard. The pronounced IGC susceptibility of the alloy led to predominantly intergranular fracture morphologies in cross-section peripheral areas after SSR testing. The results confirmed the synergistic effect among microstructure, IGC susceptibility and SCC behavior, identifying a critical window of mechanical and environmental parameters governing SCC.

## 1. Introduction

Second only to steel in use as structural metals, it is the properties combination that makes high-strength aluminum alloys versatile metallic materials for a broad range of uses where a high strength-to-weight ratio is required, including the most demanding lightweight applications in aerospace, automotive and naval sectors [1]. The 2xxx and 7xxx series containing Cu offer the best mechanical properties for these applications, as they benefit from precipitation strengthening mechanisms after appropriate heat treatments [2]. However, issues in terms of corrosion must be considered for these alloys, which can manifest with different and mutually related mechanisms, as highlighted by several studies on the corrosion behavior of Al-Cu alloys [3,4,5]. Indeed, aluminum’s good resistance to atmospheric corrosion is given by the passivity produced by a protective oxide film on its surface [6], which is stable in a broad range of pH and potentials, representing the majority of industrial environments where these alloys are used. This passive layer may deteriorate under specific environmental and loading conditions, drastically increasing corrosion rates and deteriorating component functionality [7].

Among the main environmental factors, the presence of specific chemical species—e.g., chlorides—can have a destabilizing effect on the oxide film. According to the model proposed by McCafferty [8], this process proceeds with a sequential mechanism that first involves the adsorption of Cl^-^ anions onto the oxide film surface. Its subsequent migration through the same layer leads to the localized dissolution of the metal at the metal–oxide interface [9], also leading to the formation of a strongly acidic blister, which can then break due to the evolved hydrogen gas as a result of the cathodic reduction of H+ ions. In the specific case of 2xxx alloys, this mechanism is also accompanied by the presence of an intrinsically less protective oxide film, which is locally affected by galvanic coupling with the noble precipitates rich in Cu. Furthermore, rupture of the passive layer under tensile stress conditions also leads to faster dissolution of the substrate due to direct exposure of the bare metal to the environment [10]. The interruption of the film of passivity can also lead to corrosion initiations and propagation related to the microstructural inhomogeneities represented by the aforementioned noble precipitates [11]. The presence of lattice defects at the grain boundary favors the precipitation of second phases, which can lead to micro-galvanic coupling with the surrounding α-aluminum matrix, with a typical morphology of intergranular corrosion (IGC) [12]. In the case of 2xxx aluminum alloys, the IGC susceptibility is mainly affected by the presence of θ (Al_2_Cu) and S (Al_2_CuMg) second phases, but also Al-Cu-Mn-Fe (formed during solidification), and Al-Cu-Mn (formed during homogenization) dispersoid particles [13]. All the above-mentioned phases typically exhibit a cathodic effect with respect to the aluminum matrix, which is particularly efficient in the case of Cu-containing ones [14,15]. The cathodic activity of these particles is further enhanced by the presence of a precipitate-free zone (PFZ) in the adjacent matrix, which behaves as an anode, leading to peculiar intergranular attacks.

Aluminum alloys can fail by cracking along grain boundaries when simultaneously exposed to specific environments, such as water vapor, aqueous solutions, or organic liquids, and stresses of sufficient magnitude, a phenomenon of environment-induced intergranular cracking known as stress corrosion cracking (SCC) [16]. SCC plays a major role in most of the aircraft failures known from the literature [17], but it is also relevant in other industrial fields, without evidence of the material’s damage before failure [18,19]. It is assumed that, for Al-Cu alloys, SCC occurs via an anodic dissolution mechanism [20]. Experimental studies indicate that under chloride exposure, the SCC growth rate of 2xxx alloys is highly dependent on the environment, with factors such as temperature and stress intensity influencing crack propagation [21].

Several papers report that localized attacks can trigger fatigue and/or stress corrosion cracks [22,23,24]. Residual mechanical resistance of pre-corroded specimens is usually carried out without the systematic evaluation of strain rate (SR) and relative humidity (RH) conditions. Slow strain rate (SSR) testing after pre-soaking in a chloride-rich environment over a range of imposed strain rate (SR) and relative humidity (RH) can provide full details of the environmentally assisted degradation on the mechanical response of the material [25]. During SSR testing, the continuous application of a controlled strain on the specimen facilitates crack nucleation and propagation, if the imposed strain rate is low enough to let corrosion phenomena occur alongside the mechanical stress [26]. In chloride-rich environments, SSR testing has revealed that localized pH shifts at crack tips, driven by hydrolysis reactions of dissolved aluminum ions, further destabilize the passive layer and accelerate crack growth rates [27]. Moreover, the synergistic effect of stress and electrochemical reactions can alter the local environment at the IGC tips, leading to an acceleration of the growth rate and also to catastrophic failures when an intergranular crack, subjected to a normal tensile stress, develops into IG-SCC [12,28]. The numerous factors and diverse sites that promote SCC crack initiation make it inherently difficult to prevent in structures fabricated from materials susceptible to this form of degradation. This points out the necessity to fully understand and predict the growth rate of stress corrosion cracks to ensure structural safety and desirable lifetime [16], while also assessing how different parameters interact to produce the most severe detrimental effects.

The aim of this paper is to methodically evaluate the effect of SR and RH on the SCC susceptibility and fracture morphology of an Al-Cu alloy, considering ranges representative of typical service conditions for this class of materials. In addition, the effect of pre-soaking in a chloride-containing solution was studied to assess the material’s response when localized corrosion has already been triggered. The novelty of the work mainly lies in its systematic approach to quantifying the synergic effect of these key parameters on SCC susceptibility, as it cannot be directly predicted from the superposition of the effects of single parameters.

## 2. Materials and Methods

### 2.1. Material

The investigated Al-Cu alloy is a commercial AW 2017A in T4 condition, provided by the supplier (Aviometal S.p.A., Arsago Seprio, Varese, Italy) as a 2000 mm × 1000 mm × 4 mm sheet metal. The chemical composition of the alloy is reported in Table 1. The manufacturing process and the performed heat treatment resulted in a Vickers hardness of 127.3 ± 4.1 HV1, assessed with 25 indentations according to the ISO 6507 standard [29]. The thermo-mechanical route adopted for the studied alloy is commonly used to improve mechanical resistance but can also lead to IGC susceptibility.

### 2.2. Microstructural Investigation

The microstructure of 2017A-T4 alloy was evaluated on 20 mm × 20 mm × 4 mm specimens, by preparing metallographic sections along the principal material directions, namely longitudinal (L), long-transverse (LT) and short-transverse (ST). The cross sections were mechanically ground with SiC emery papers up to 4000 grit and subsequently polished with a 1 µm diamond suspension. The microstructure was highlighted via chemical etching using Keller’s reagent (5 mL HNO_3_, 3 mL HCl, 2 mL HF, 190 mL distilled water), according to the ASTM E407 standard [30]. Microstructural investigations were performed using a Keyence VHX-7100 (Keyence Italia S.p.A., Milan, Italy) digital-optical microscope (OM) and a Zeiss Sigma 300 (Carl Zeiss S.p.A., Milan, Italy) field-emission scanning electron microscope (FESEM), equipped with an Oxford X-Act probe for energy dispersive X-ray spectroscopy (EDS).

### 2.3. Corrosion Behavior Assessment

#### 2.3.1. Intergranular Corrosion Susceptibility Test

Susceptibility to selective corrosion was assessed according to the ISO 11846 (method B) standard [31]. Two specimens with similar geometry to those used for microstructural investigation were tested, after mechanically grinding all the exposed surfaces with SiC emery papers up to 4000 grit. The specimens were measured with a Vernier caliper, then pickled in 8 wt.% NaOH solution at 55 °C for 4 min and immersed in concentrated HNO_3_ (65 wt.%) for 2 min. The samples were then weighed with an analytical balance (sensitivity 0.00001 g), performing 3 measurements for each specimen. The specimens were finally immersed in the test solution (30 g/L NaCl + 10 mL/L conc. HCl) at 25 ± 1 °C for 24 h. After immersion, the specimens were rinsed in distilled water and dried. Corrosion products were removed with a non-metallic brush while rinsing in distilled water. The samples were then immersed in an ultrasonic bath of acetone and subsequently allowed to dry. The mass loss during the test was obtained as the difference between the average initial mass of the specimen and that measured after the immersion period. The average corrosion rate was calculated as a quantitative indicator of IGC phenomena. At the end of the test, metallographic sections were obtained to assess the presence, morphology, and depth of the corrosion attacks with OM and FESEM.

#### 2.3.2. Volta Potential Measurements

Scanning Kelvin probe force microscopy (SKPFM) analyses were performed on polished, unetched specimens using a Park Nanoscope III (Park Systems Europe GmbH, Mannheim, Germany) multimode atomic force microscope (AFM) to investigate the effect of macro precipitates on corrosion behavior by analyzing the Volta potential distribution. Topographic and Volta potential maps in the 50 × 50–100 × 100 µm^2^ range were acquired employing n+-silicon tips coated with PtIr, setting a 0.05 Hz scan frequency. The surface Volta potential was measured in lift mode with a scan height of 100 nm.

#### 2.3.3. Tensile and SSR Tests

The tensile tests and the SSR tests were carried out using dog-bone-shaped flat specimens with a 4 × 8 mm^2^ resistant cross-section, 43 mm parallel length, and 32 mm gauge length, compliant with the ISO 6892-1 standard [32]. Before the tensile and SSR tests, the specimen surfaces were ground with SiC abrasive papers up to 1200 grit, degreased with acetone, and stored in a dryer with silica gel. Some specimens were then subjected to a pre-soaking in a 3.5 wt.% NaCl solution at 25 ± 1 °C for 1 week (168 h). The selected pre-soaking duration was defined based on a previous study by Holroyd et al. on an AA 2024 alloy, which demonstrated a significant effect of pre-soaking in 0.53 M NaCl (approx. 3.1 wt.%) solution for exposure times up to 300 h [28]. Solutions with similar chloride concentration can be considered representative of marine environments, to which these alloys may be exposed in real operational scenarios. The cross-sections of corrosion attacks that occurred during the pre-corrosion treatment were also investigated at OM and FESEM. The tensile tests were carried out using a Galdabini Sun 5 (Galdabini S.p.A., Varese, Italy) universal testing machine equipped with a 50 kN load cell. These tests were performed in laboratory environment conditions, setting an SR of 10^−3^ or 10^−4^ s^−1^. The SSR tests were carried out using a custom-made testing machine with four independent loading stations, each equipped with a 30 kN load cell. External devices have been fitted to the testing machine to monitor and control the temperature and RH within the test chamber. The tests were carried out at 25 °C with SR of 10^−5^, 10^−6^, and 10^−7^ s^−1^ and RH of 40%, 60% and 80%. Such ranges cover many of the environmental and loading conditions to which high-strength aluminum alloy components, typically exposed to atmospheric environments, may be subjected. The test temperature and RH were maintained within ± 2 °C and ± 5%, respectively. SR was calculated by dividing the crosshead speed by the initial parallel length. The gauge length and the dimensions of the resistant section were measured before and after each test by an optical meter (0.01 mm resolution) to estimate the elongation at break (A%) and reduction in area (Z%), according to Equations (1) and (2).

(1) $$A\%=\frac{\left(L_{f}-L_{i}\right)}{L_{i}}\;\times \;100$$

(2) $$Z\%=\frac{\left(A_{f}-A_{i}\right)}{A_{i}}\;\times \;100$$

where L_i_ and L_f_ are the initial and final gauge length, and A_i_ and A_f_ are the initial and final cross-sectional areas.

## 3. Results and Discussion

### 3.1. Microstructural Investigation and IGC Susceptibility

**Figure 1:**
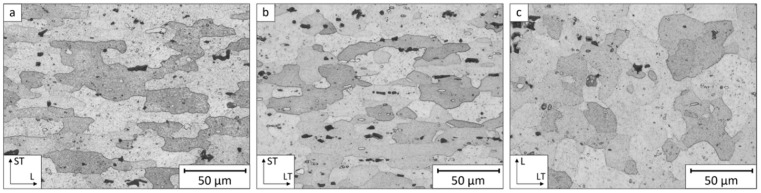
Low-magnification OM micrographs of the 2017A-T4 microstructure along (**a**) L-ST, (**b**) LT-ST, and (**c**) LT-L directions (etching solution: Keller’s reagent).

The microstructure of 2017A-T4 alloy, shown in Figure 1, consisted of elongated α-Al grains oriented along the LT and, more prominently, the L direction, as typically observed in rolled alloys of the 2xxx series [33]. The grain aspect ratio, as a result of the deformation induced by rolling, was retained even after heat treatment, although the high temperatures reached during the annealing stage likely promote partial recrystallization and grain coarsening [34].

**Figure 2:**
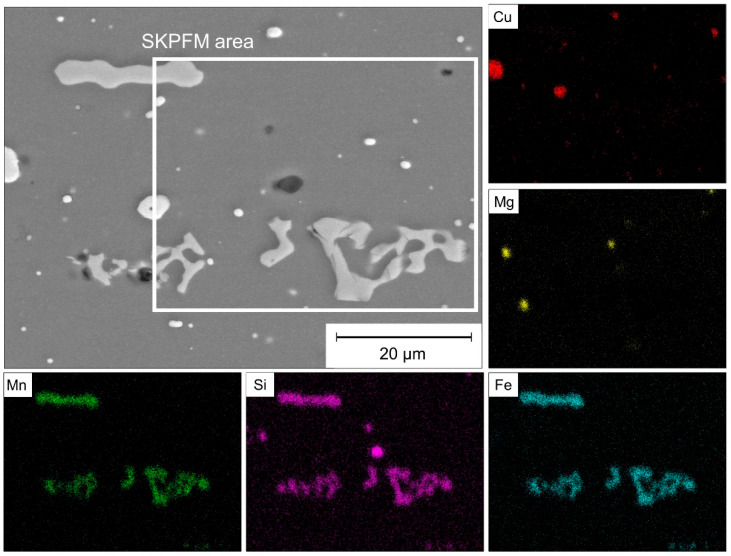
FESEM micrographs and EDS maps of the different secondary phases observed in the 2017A-T4 alloy.

The presence of secondary phases was also evidenced after chemical etching by the presence of several black spots. Coarser secondary phases were slightly aligned along the rolling direction, while smaller phases were found both at grain boundaries and within grains. Several studies have demonstrated through X-ray diffraction (XRD) analysis the formation of Cu-rich precipitates—namely Al_2_Cu and Al_2_CuMg—for similar heat-treated alloys [33,34,35]. These hard precipitates, when nanometric and finely dispersed, are primarily responsible for the high mechanical strength of Cu-containing aluminum alloys, enabling the precipitation hardening mechanism [36]. However, they may occasionally appear with sizes in the order of a few micrometers, due to their coalescence and/or incomplete dissolution within the matrix during solubilization treatment. The presence of coarse and almost spherical Cu-rich precipitates was also confirmed in the investigated 2017A-T4 alloy, as evidenced by further FESEM-EDS analysis of the secondary phases, shown in Figure 2. In addition, Mg- and Si-rich globular secondary phases were detected, most likely corresponding to Mg_2_Si precipitates [34]. Although these precipitates are typically found in Al-Mg-Si aluminum alloys (6xxx series), they can also form in 2xxx series alloys containing low amounts of such elements (Table 1). Finally, coarser precipitates were identified as AlFeMnSi intermetallic, as already reported by Huang et al. [34], who performed transmission electron microscopy (TEM) analyses on the same alloy. In the case of Mg-Si and AlFeMnSi precipitates, their coarse size—in the order of a few micrometers and several tens of micrometers, respectively—is also likely attributable to incomplete dissolution during the solubilization heat treatment.

**Figure 3:**
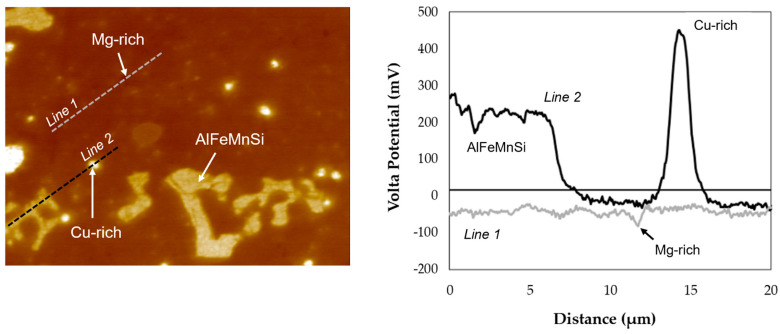
Volta potential analysis of the second phases showed in Figure 2.

Coarse secondary phases in Figure 2 were further investigated with SKPFM measurements, highlighting the Volta potential distribution shown in Figure 3. Cu-rich precipitates exhibited the highest Volta potential values, with a difference of approximately +410 mV compared to the adjacent α-Al matrix. This result is consistent with the findings reported by Zhang et al. [37] on Al-Zn-Mg-(Cu) alloys, which showed larger local variations in corrosion potential with increasing Cu content, and from those seen in LPBF-produced aluminum–copper alloys from Lorenzi et al. [38]. Conversely, the AlFeMnSi intermetallic exhibited a lower Volta potential, although higher than that of the aluminum matrix, with potential differences of approximately +218 mV. Finally, the Mg-Si precipitates showed the lowest Volta potential, even slightly below that of the α-Al phase by approximately −23 mV. Several studies have demonstrated that the Volta potential can be correlated with the nobility of the phases present in aluminum alloys. Accordingly, the SKPFM-measured potential values allow for the prediction of possible micro-galvanic coupling among the different phases observed in the 2017A-T4 alloy. It is widely reported that the Cu-rich phases behave as highly noble particles [37], also exhibiting high cathodic efficiency toward the oxygen reduction reaction (ORR). In contrast, the AlFeMnSi intermetallic exhibited a lower driving force for galvanic coupling with the surrounding aluminum matrix. However, their potential differences with respect to the aluminum matrix are not negligible, and their large size may further exacerbate the phenomenon. Finally, the Mg-Si particles likely exhibit an opposite behavior, acting as local anodes with respect to the aluminum grains, due to the remarkably low nobility of magnesium [39].

**Figure 4:**
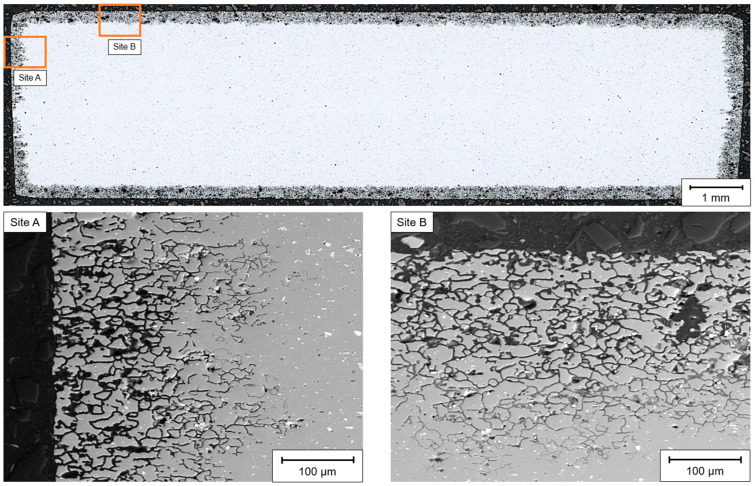
Metallographic section of IGC-tested 2017A-T4 specimen along L-ST plane at the OM, and higher magnification FESEM images of IGC attacks along (Site A) L and (Site B) ST directions.

A pronounced susceptibility of the 2017A-T4 alloy to IGC was observed (Figure 4), as expected for Al-Cu alloys [40]. The average corrosion rates, used as a quantitative indicator of the selective attack magnitude, reached relatively high values (1233 ± 22 mg/dm^2^·day), confirming the presence of severe IGC attacks with sporadic occurrence of grain dropping. Cross-sectional analyses confirmed the cathodic role of coarse Al-Cu and AlFeMnSi precipitates, which were embedded within corrosion products at sites of selective attack. However, it is important to note that thin IGC attacks also occurred in the absence of such coarse precipitates, likely promoted by nanometric secondary phases precipitated along grain boundaries, as extensively documented in the literature for heat-treated wrought Al-Cu alloys [41,42]. A comparable propagation of IGC attacks in both the L and ST directions, with a maximum penetration depth of 396 ± 62 µm and 358 ± 27 µm, respectively.

### 3.2. SCC Behavior and the Role of Pre-Soaking

**Figure 5:**
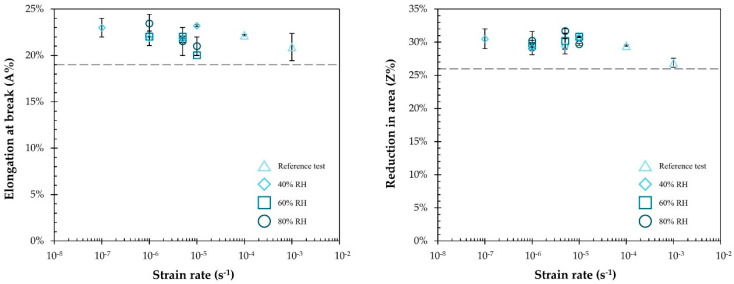
Elongation at break (A%) and reduction in area (Z%) of not pre-soaked specimens (dashed lines represent lower A% and Z% thresholds of not pre-soaked specimens).

**Figure 6:**
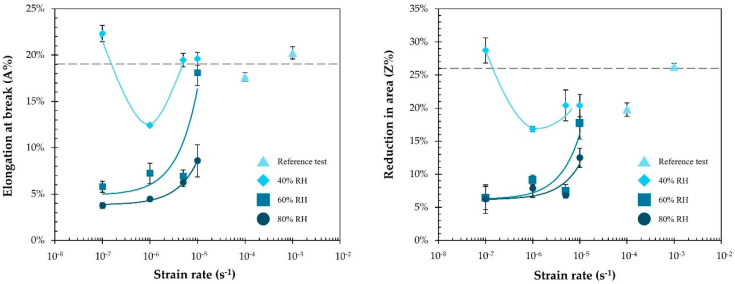
Elongation at break (A%) and reduction in area (Z%) of pre-soaked specimens (dashed lines represent lower A% and Z% thresholds of not pre-soaked specimens).

Engineering stress–engineering strain curves were used to extract synthetic parameters suitable for quantifying the alloy susceptibility to SCC. Parameters such as yield strength (YS) and ultimate tensile strength (UTS) proved to be poorly sensitive in discriminating among the different conditions. Conversely, ductility-related indices such as elongation at break (A%) and reduction in area (Z%), showed greater effectiveness, in agreement with several studies reported in the literature [43,44]. The evolution of these parameters as a function of SR and RH is shown in Figure 5 and Figure 6 for the not pre-soaked and pre-soaked specimens, respectively. The values of A% and Z% parameters for each testing condition, as well as the experimental engineering stress–engineering strain curves, are reported in Appendix A.

For specimens that were not pre-soaked in 3.5 wt.% NaCl solution, A% and Z% were essentially comparable across all testing conditions (Figure 5). This behavior confirmed the absence of SCC susceptibility in not pre-soaked 2017A-T4, despite the application of a constant SR, which continuously disrupts the passive film through mechanical deformation. According to the slip-dissolution mechanism at the crack tip, these results indicate that the mere oxide film rupture is not sufficient to establish sustained preferential attack at an active crack tip supported by passive crack walls and external surfaces, even at relatively high RH and low SR. This is likely ascribable to the highly insulating nature of aluminum oxides [45], which can limit the effectiveness of such SCC mechanisms.

Conversely, pre-soaked specimens showed a strong detrimental effect of both SR and RH on A% and Z% (Figure 6). The occurrence of SCC in the pre-soaked specimens can be attributed to mechanisms that extend beyond the rupture of the oxide film, which also takes place in the not pre-soaked specimens.

**Figure 7:**
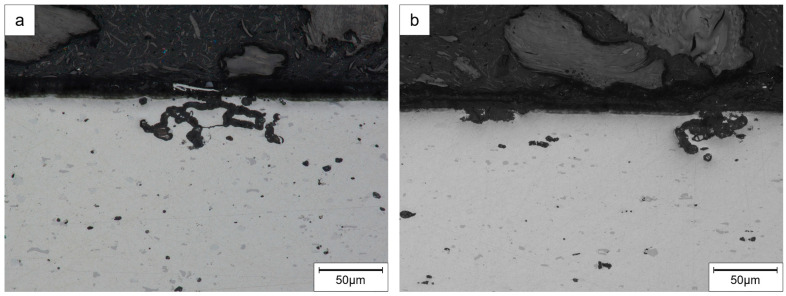
OM micrograph of different corrosion attacks morphologies after pre-soaking: (**a**) branched/intergranular and (**b**) localized.

Indeed, following pre-soaking, corrosion attacks with penetration depths in the tens of microns range were observed (Figure 7), exhibiting a mixed morphology attributable to both localized corrosion and selective intergranular attack.

**Figure 8:**
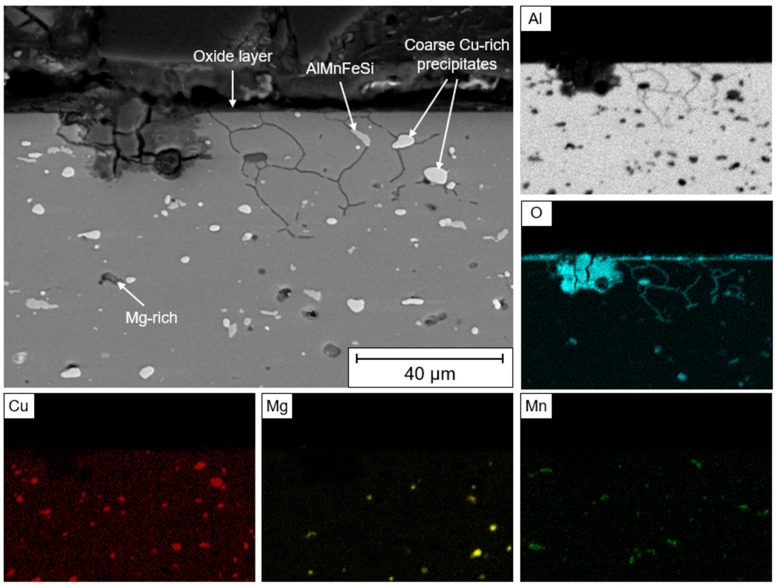
FESEM micrograph and EDS analysis on a metallographic cross-section of a pre-soaked specimen.

The pre-soaking was carried out in a chloride-containing solution with a neutral pH. Under near-neutral conditions, aluminum alloys generally achieve passive behavior through the formation of a protective oxide film. However, this film can be locally destabilized by chloride ions, which are initially adsorbed onto the oxide film and subsequently migrate toward the metal–oxide interface, where they promote the development of localized corrosion attacks. This process does not involve the entire exposed surface but instead manifests as spatially separated pits. Once a pit nucleates and creates a discontinuity in the passive film, the local environment within the localized attack may differ markedly from that of the bulk electrolyte. Indeed, the development of localized corrosion is known to promote strong local acidification, due to metal anodic dissolution and subsequent metal ion hydrolysis. For instance, in the case of pure aluminum, McCafferty estimated pH values within the localized attack blister in the range of 0.85 to 2.3 [8]. Under such environmental conditions, the corrosion front may propagate either uniformly or along preferential paths dictated by the microstructure, which manifest due to micro galvanic coupling between different phases. Al-Cu and AlFeMnSi coarse secondary phases still exhibited a cathodic activity during pre-soaking, promoting preferential dissolution of the adjacent aluminum matrix, and they were consistently found in the proximity of the corroded regions (Figure 8). Even after pre-soaking, narrow corrosion attacks associated with nanoscale grain-boundary precipitates were observed to propagate even in the absence of macro-precipitates. Moreover, abundant corrosion products were detected within the cavities generated during pre-soaking, as indicated by the local oxygen enrichment revealed by the EDS analyses.

The presence of corrosion products after pre-soaking, resulting from the near-neutral pH of the solution which promotes their reprecipitation, is particularly relevant for atmospheric corrosion phenomena, including SCC tests conducted in humid air, as they play a crucial role in governing moisture condensation and thereby provide the medium necessary for electrochemical reactions to proceed. The hygroscopic nature of aluminum corrosion products facilitates water condensation even at relatively low RH through both physical and chemical condensation mechanisms. This surface condensate typically consists of more than a single adsorbed water monolayer, even under moderately elevated humidity, and is only partially described by classical Langmuir-type adsorption phenomena, as it also partially fills the surface micropores present within the outer oxide layer [46].

Localized corrosion attacks are electrochemically active material regions, where corrosion attacks preferentially propagate. SCC cracks advancement in these regions is therefore governed not only by mechanical stress intensification but also by the synergistic interaction between mechanical loading and corrosion reactions. As described by the mechano-chemical theory proposed by Gutman [47], the stress concentration at the crack tip—where local plastic deformation occurs—increases the internal energy of the system, thereby altering both the electrode potential and the activation energy of anodic dissolution reactions. Under these conditions, applied stress effectively acts as a chemical driving force, mechanically assisting material dissolution. Furthermore, the presence of a relatively unstable oxide film within the localized corrosion sites—resulting from the combined effects of mechanical stress and the acidic local environment—may promote the adsorption and subsequent ingress of monoatomic hydrogen, which is well known to induce embrittlement in Al alloys [48].

The A% and Z% values of the pre-soaked specimens tested under conditions less favorable for SCC initiation (40% RH, 10^−4^÷10^−3^ s^−1^ SR) were only slightly lower than those measured for the not pre-soaked specimens tested at the same RH and SR conditions. This observation suggested that the mere intensification of local stress associated with the localized/IGC attacks formed during the pre-soaking stage (Figure 7 and Figure 8) played only a marginal role in reducing the material deformability. In other words, under the adopted experimental conditions, the failure mechanism can be primarily attributed to SCC rather than to corrosion-induced mechanical property degradation (CIMPD) resulting from the presence of localized corrosion attacks. This conclusion holds despite the fact that CIMPD has been documented in several aluminum alloys as a potentially relevant contribution, both through material degradation and stress state alteration, as well as through effects associated with absorbed hydrogen generated by corrosion processes [49].

**Figure 9:**
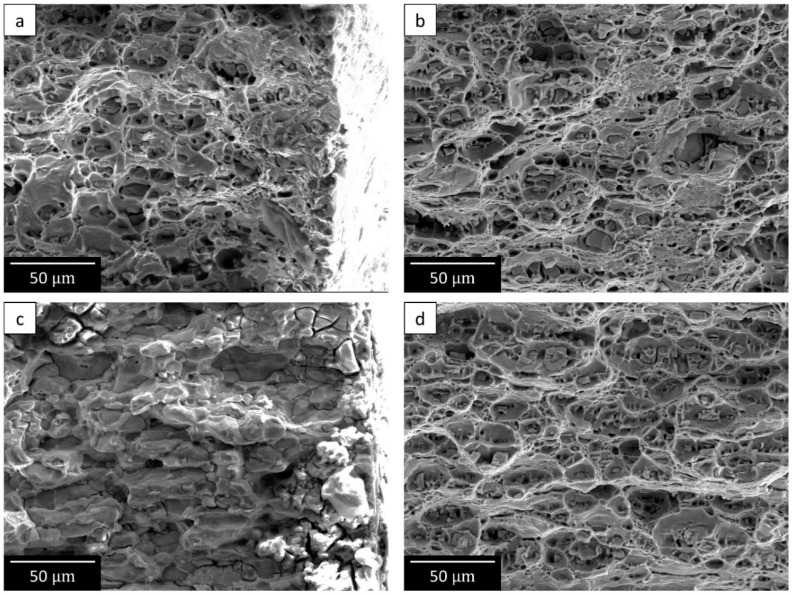
FESEM analysis of fracture surfaces of (**a**,**b**) not pre-soaked and (**c**,**d**) pre-soaked specimens in peripheral and core regions, respectively (testing conditions: 80% RH, 10^−6^ s^−1^ SR).

These results were further validated by comparing the fracture surfaces of not pre-soaked and pre-soaked specimens tested under identical conditions (10^−6^ s^−1^ SR, 80% RH). Figure 9 shows representative micrographs of the characteristic fracture morphologies observed in the peripheral and central regions of such fracture surfaces. The selected testing conditions correspond to the SR and RH combination that produced one of the most pronounced differences between not pre-soaked and pre-soaked specimens. The not pre-soaked specimen exhibited a fracture surface characterized by dimples in both the peripheral (Figure 9a) and central regions (Figure 9b). This morphology indicates a predominantly ductile fracture mechanism, consistent with void nucleation, growth, and coalescence, which is typically observed in the absence of environmentally induced degradation [50]. Conversely, the pre-soaked specimens exhibited a mixed-mode fracture morphology, with a clear macroscopic distinction between the peripheral and core regions of the load-bearing cross section. The peripheral regions of the cross sections were characterized by step-like facets and crack paths propagating along grain boundaries, decorated with corrosion products (Figure 9c). These features are characteristic of intergranular stress–corrosion cracking (IG-SCC) and are commonly reported in heat-treatable Al-Cu alloys, where Cu-rich precipitates at the grain boundaries increase susceptibility to intergranular attack, as also confirmed by the immersion tests according to ISO 11846. In contrast, the central region maintained a ductile fracture morphology, featuring dimples similar to those observed in the not pre-soaked specimens, consistent with a failure mechanism governed primarily by mechanical deformation (Figure 9d).

**Figure 10:**
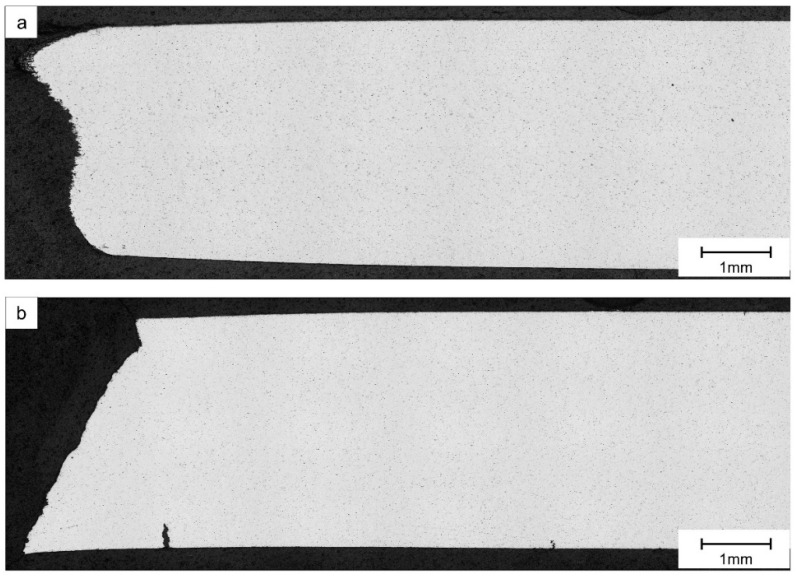
Metallographic sections parallel to the loading direction of SSR specimens (40% RH, 10^−6^ s^−1^ SR): (**a**) non-pre-soaked and (**b**) pre-soaked.

Finally, metallographic cross-sections taken along the loading direction further corroborated the corrosion mechanism and the differences observed between not pre-soaked and pre-soaked conditions (Figure 10). Not pre-soaked specimens exhibited pronounced necking near the fracture surface (Figure 10a), consistent with extensive plastic deformation before failure. Conversely, pre-soaked specimens showed only a negligible reduction in the resistant cross-section in proximity of the main fracture surface, alongside multiple secondary cracks (Figure 10b). Secondary cracks are characteristic of SCC-related damage [51,52] and support the interpretation that environmentally assisted mechanisms governed the failure process in the pre-soaked condition.

Overall, fracture morphology confirmed the occurrence of SCC in pre-soaked 2017A-T4 alloy subjected to SSR testing under humid conditions, with a dominant intergranular mechanism at the surface and a gradual transition to ductile failure in the unaffected central region. This gradient is consistent with literature on SCC in Al-Cu alloys and underscores the primary role of microstructural–environment interactions in governing crack initiation and propagation [53].

### 3.3. Effect of SR and RH on SCC Susceptibility

A stress corrosion cracking index (SCC_index_) was introduced to quantify SR and RH effects by comparing the A% of pre-soaked specimens with that of reference, non-pre-soaked specimens (A%_ref_) tested at the same conditions, as defined in Equation (3):

(3) $${\mathrm{SCC}}_{\mathrm{index}}\;=\;\left(1\;-\frac{A\%}{{A\%}_{\mathrm{ref}}}\right)\;\times \;100$$

This index enables normalization of the reduction in ductility observed under SCC-promoting conditions, compensating for the slight variations measured in the not pre-soaked specimens as a function of SR. For the calculation of this index, elongation at fracture (A%) was used as the ductility parameter instead of reduction in area (Z%). This choice was dictated by the specimen geometry, since the rectangular cross-section does not allow uniform necking in different directions and is influenced by edge effects, thereby reducing the reliability of Z% in describing the material ductility.

The results confirmed the previous observations and further highlighted the influence of the specific testing parameters–i.e., SR and RH–on SCC susceptibility.

**Figure 11:**
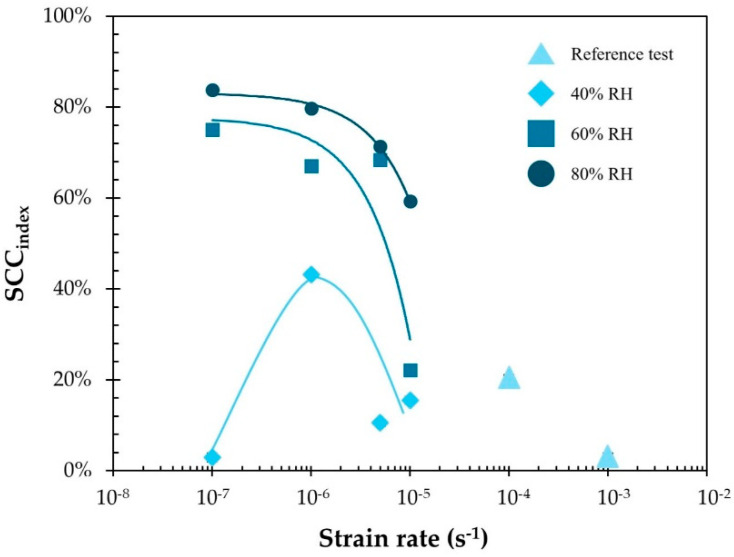
SCC susceptibility index as a function of SR and RH.

Figure 11 shows the evolution of the SCC index as a function of SR and RH. The decrease in SR generally led to an increase in SCC susceptibility. Under slower deformation, the electrochemical reactions involved in crack propagation have more time to progress before the crack advances through mechanisms primarily driven by mechanical stress intensification. The correlation between SR and the SCC index was found to be strongly non-linear, with a pronounced increase in SCC susceptibility observed for strain rates below approximately 10^−5^ s^−1^. Similarly, increasing RH significantly enhanced SCC susceptibility at a given SR, promoting the formation of surface condensate, which provides the medium necessary for corrosion reactions. The most marked effect was observed when RH increased from 40% to higher levels.

An exception was observed at 40% RH, where a very low strain rate (10^−7^ s^−1^) led to a reduction in SCC susceptibility, as evidenced by a decrease in the SCC index. This exception supports the stress-assisted crack tip dissolution mechanism; nonetheless, hydrogen embrittlement may still play a role. Indeed, the adsorption of hydrogen within the material is plausible under the unique local conditions formed inside the corrosion sites–namely acidic pH and a destabilized oxide film. Unlike conventional hydrogen embrittlement, in this case, hydrogen is supplied primarily at the propagating crack front rather than throughout the surrounding metal, which remains passivated. Therefore, it cannot be excluded that SCC propagation in this alloy occurs through a mixed mechanism combining the theoretical models proposed, despite the generally low diffusivity of hydrogen in aluminum [48,54]. It is also worth noting that the reduced SCC susceptibility at 40% RH for SR below 10^−6^ s^−1^ may additionally be attributed to surface drying due to the long duration of the test. Prolonged exposure at such low RH can cause a significant loss of condensate at the crack tip, sufficiently hindering the electrochemical reactions involved [28].

**Figure 12:**
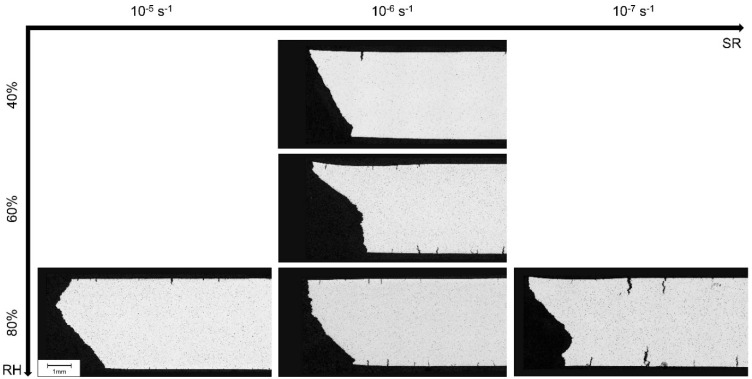
Metallographic sections parallel to the loading direction of SSR specimens at different imposed SR and RH.

Overall, the observed increase in SCC susceptibility with decreasing SR and increasing RH is consistent with the metallographic evidence. Cross-sections taken along the loading direction for different SR and RH conditions (Figure 12) clearly showed the progressive intensification of SCC-related features, including reduced necking and a higher density of secondary cracks.

**Figure 13:**
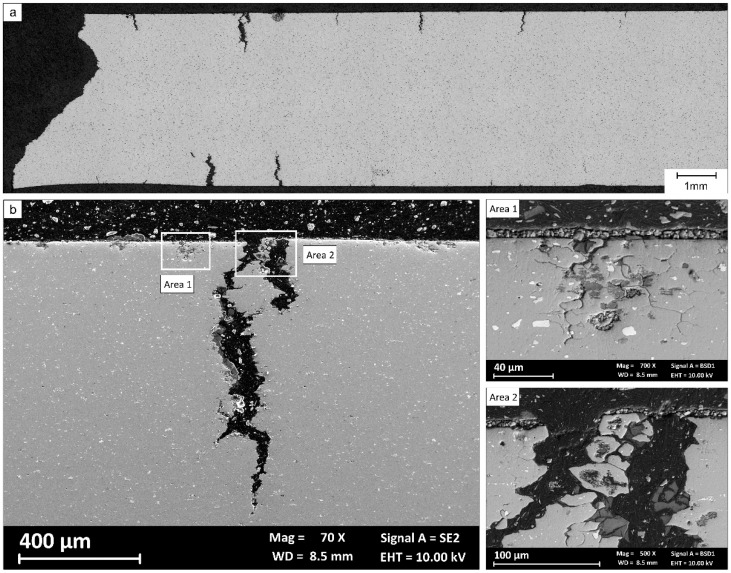
(**a**) OM and (**b**) FESEM analyses of a secondary crack (pre-soaked specimen, 10^−7^ s^−1^ SR, 80% RH).

Finally, the analysis of secondary cracks provided further evidence of the synergy between IGC and SCC susceptibility. To this end, the specimen tested under the most aggressive conditions (pre-soaked, 10^−7^ s^−1^ SR, 80% RH) was considered. FESEM observations of secondary cracks in Figure 13 revealed clear grain-boundary selective attack. This was observed both in larger cracks, where SCC propagation also led to crack widening, and in the smaller cracks, where the attack appeared as thin, filament-like attacks, closely resembling those produced during the IGC immersion tests. As the main cracks propagate, a localized plastic deformation develops at the crack tip, which leads to an effective widening of the crack tip. This, in turn, facilitates the branching of SCC attack along the preferred grain-boundary paths, due to the already mentioned, microstructure-related mechanisms. It is important to note that this effect is not observed in pre-soaked specimens tested at low RH, nor in not pre-soaked specimens at high RH, confirming that SCC phenomena require the combined presence of a localized attack and an environment that maintains sufficient moisture at the crack tip to support crack propagation.

## 4. Conclusions

Within this study, the influence of pre-soaking, SR and RH on the SCC susceptibility of an aluminum–copper alloy was investigated. Based on the experimental results, the following conclusions can be drawn:

- In the absence of pre-existing localized attack—e.g., those promoted by exposure to chloride-containing solutions—the material did not exhibit any susceptibility to SCC, regardless of RH or applied SR; this happens despite continuous mechanically induced rupture of the passive film.
- A significant increase in SCC susceptibility was observed after pre-soaking in a chloride-containing solution, leading to losses in ductility up to 84%. The presence of specific environmental conditions within localized corrosion sites acted as a necessary location for crack initiation during subsequent SSR testing.
- The morphology of corrosion attacks, fracture surfaces, and secondary cracks confirmed the intergranular nature of SCC propagation in the studied alloy; the failure mode progressively transitioned from intergranular fracture at the specimen surface to ductile fracture in the central region, indicating a final failure stage predominantly governed by mechanical factors.
- A pronounced dependence on both SR and RH was observed for pre-soaked specimens. SCC susceptibility increases markedly at SRs equal to or lower than 10^−^^5^ s^−^^1^, and RHs above 60% further exacerbate the phenomena, with ductility losses in any case higher than 11%.

The obtained results provide a robust framework for assessing SCC behavior in high-strength aluminum alloys and may be effectively extended to novel aluminum alloys produced through advanced manufacturing routes, where unconventional microstructures may further influence environmentally assisted cracking behavior. Moreover, the adopted methodology and the ranges of SR and RH considered can be regarded as representative of a wide range of engineering applications involving high-strength Al alloys. These range from defect propagation in structural components for the aeronautical and aerospace sectors to other lightweight components exposed to atmospheric conditions.

## Figures and Tables

**Table 1:** Chemical composition (wt.%) of the investigated alloy.

Element	Al	Cu	Si	Mn	Mg	Fe	Zn	Ti	Cr
2017A	bal.	4.2	0.74	0.70	0.69	0.20	0.19	0.04	0.03

**Figure A1:**
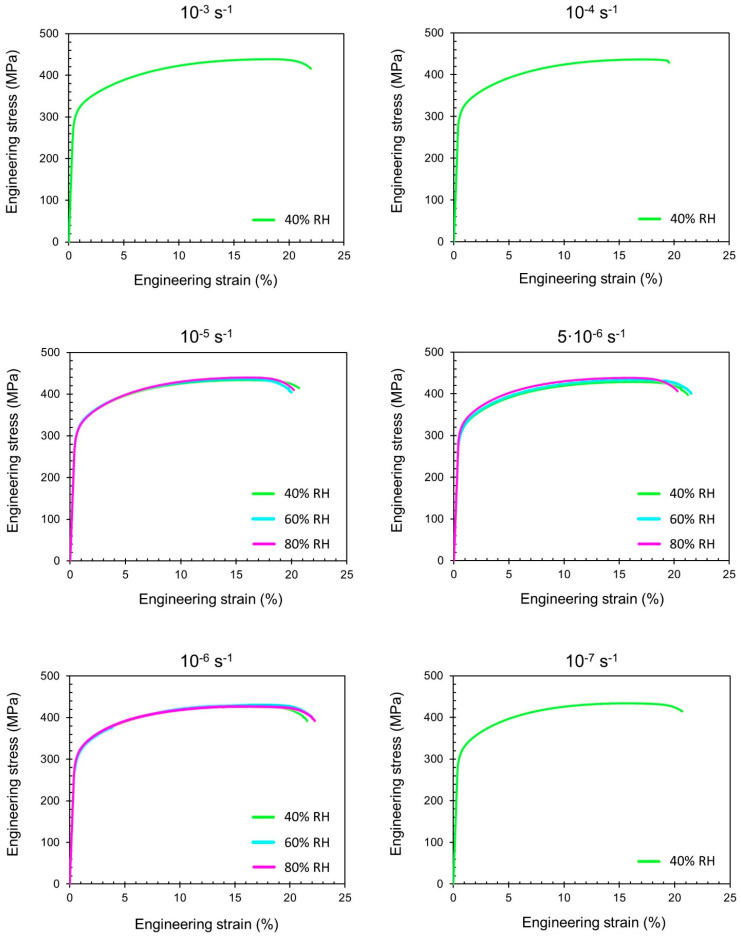
Representative stress–strain curves of not pre-soaked specimens for different SR and RH combinations.

**Figure A2:**
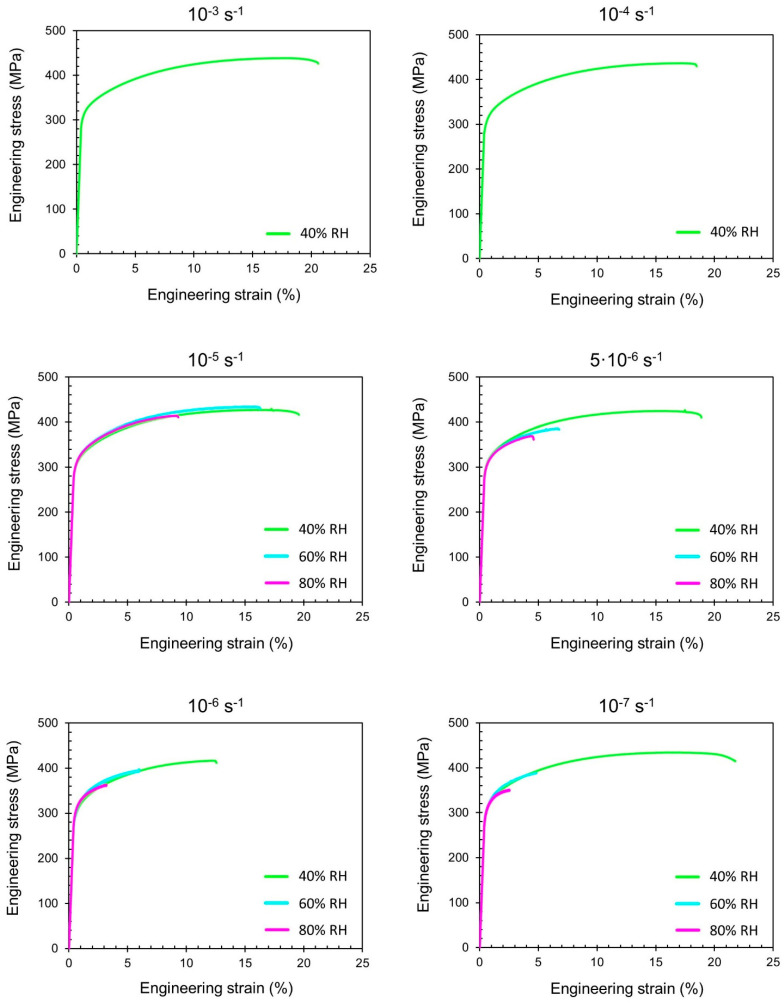
Representative stress–strain curves of pre-soaked specimens for different SR and RH combinations.

## Data Availability

The original contributions presented in this study are included in the article. Further inquiries can be directed to the corresponding author.

## Appendix A

This section reports the engineering stress–engineering strain curves for not pre-soaked (Figure A1) and pre-soaked specimens (Figure A2), and the values of A%, Z% and SCC index for all the tested conditions (Table A1).

**Table A1:** A%, Z% and SCC index of all the tested conditions.

Pre-Soaking	Strain Rate (SR)	Relative Humidity (RH)	A% (avg. ± st. dev.)	Z% (avg. ± st. dev.)	SCC_index_
Not pre-soaked	10^−3^ s^−1^	40%	20.9 ± 1.5	26.9 ± 0.7	-
		60%	-	-	-
		80%	-	-	-
	10^−4^ s^−1^	40%	22.2 ± 0.1	29.5 ± 0.1	-
		60%	-	-	-
		80%	-	-	-
	10^−5^ s^−1^	40%	23.2 ± 0.1	30.7 ± 0.1	-
		60%	20.0 ± 0.2	30.8 ± 0.2	-
		80%	21.0 ± 1.0	29.7 ± 0.1	-
	5·10^−6^ s^−1^	40%	21.7 ± 0.4	29.4 ± 1.2	-
		60%	22.0 ± 0.1	30.2 ± 1.3	-
		80%	21.5 ± 1.5	31.7 ± 0.4	-
	10^−6^ s^−1^	40%	21.9 ± 0.8	29.2 ± 1.1	-
		60%	22.0 ± 0.3	29.4 ± 0.2	-
		80%	23.4 ± 0.5	30.2 ± 1.5	-
	10^−7^ s^−1^	40%	23.0 ± 1.0	30.5 ± 1.5	-
		60%	-	-	-
		80%	-	-	-
Pre-soaked	10^−3^ s^−1^	40%	20.2 ± 0.7	26.3 ± 0.5	3%
		60%	-	-	-
		80%	-	-	-
	10^−4^ s^−1^	40%	17.6 ± 0.5	19.8 ± 1.0	21%
		60%	-	-	-
		80%	-	-	-
	10^−5^ s^−1^	40%	19.6 ± 0.7	20.4 ± 1.7	8%
		60%	18.1 ± 1.4	17.7 ± 2.4	15%
		80%	8.6 ± 1.7	12.5 ± 1.4	60%
	5·10^−6^ s^−1^	40%	19.5 ± 0.7	20.4 ± 2.3	11%
		60%	6.9 ± 0.7	7.5 ± 1.0	68%
		80%	6.2 ± 0.5	6.8 ± 0.4	71%
	10^−6^ s^−1^	40%	12.4 ± 0.1	16.9 ± 0.4	45%
		60%	7.2 ± 1.1	9.1 ± 0.7	68%
		80%	4.5 ± 0.1	7.8 ± 1.3	80%
	10^−7^ s^−1^	40%	22.3 ± 0.9	28.7 ± 1.9	3%
		60%	5.8 ± 0.6	6.4 ± 1.7	75%
		80%	3.8 ± 0.3	6.2 ± 2.2	84%
